# Impact of hyperglycemia on tuberculosis treatment outcomes: a cohort study

**DOI:** 10.1038/s41598-024-64525-3

**Published:** 2024-06-12

**Authors:** Xu Yanqiu, Yang Yang, Wu Xiaoqing, Lei Zhixuan, Zhao Kuan, Guo Xin, Zhang Bo, Wang Jinyu, Cai Jing, Ma Yan, Ma Aiguo

**Affiliations:** 1 Institute of Nutrition and Health, School of Public Health, Qingdao University, Qingdao, Shandong China; 2 Yuncheng Center for Disease Control and Prevention, Yuncheng, Shanxi China; 3 Qingdao No.6 People’s Hospital, Qingdao, Shandong China; 4 Department of Infection and Disease Control, Sunshine Union Hospital, Weifang, Shandong China; 5 Weifang No.2 People’s Hospital, Weifang, Shandong China

**Keywords:** Infectious diseases, Tuberculosis

## Abstract

Hyperglycemia is prevalent and closely associated with pulmonary tuberculosis (PTB). This study aimed to investigate the effects of hyperglycemia on the outcomes of PTB treatment. This study comprised 791 patients with PTB in total. Patients with fasting plasma glucose levels of ≥ 6.1 mmol/L were diagnosed with hyperglycemia. Anthropometric and baseline demographic data were also collected. The treatment response was assessed based on clinical symptoms (sputum production, cough, chest pain, fever, hemoptysis, night sweats, loss of appetite, and fatigue), sputum smear, chest computed tomography (CT), and adverse gastrointestinal responses (vomiting, nausea, abdominal distension, diarrhea, and constipation). A generalized estimating equation (GEE) was used to evaluate these relationships. Hyperglycemia affected 266 (33.6%) of the 791 patients with PTB. In GEE analyses, patients with hyperglycemia exhibited a greater incidence of elevated tuberculosis (TB) scores (odds ratio (OR) 1.569; 95% CI 1.040–2.369), cough (OR 1.332; 95% CI 1.050–1.690), and night sweats (OR 1.694; 95% CI 1.288–2.335). Hyperglycemia was linked with a higher risk of positive sputum smears (OR 1.941; 95% CI 1.382–2.727). During therapy, hyperglycemia was also associated with an increased incidence of vomiting (OR 2.738; 95% CI 1.041–7.198), abdominal distension (OR 2.230; 95% CI 1.193–4.171), and constipation (OR 2.372; 95% CI 1.442–3.902). However, the CT results indicated that hyperglycemia did not affect pulmonary lesions in patients with TB. Patients with TB and hyperglycemia are at a higher risk of severe clinical manifestations, positive sputum smears, and adverse gastrointestinal effects and, therefore, the special situation of hyperglycemic patients should be considered in the prevention and treatment of TB.

## Introduction

Tuberculosis (TB) is a highly contagious disease caused by the bacteria *Mycobacterium tuberculosis* (*M. tuberculosis*). It remains a major global health concern, ranking among the leading causes of morbidity and mortality globally^1^. TB is the second-highest leading cause of death from a single infectious agent after SARS-CoV-2 which caused the COVID-19 pandemic^2^. According to the World Health Organization (WHO), 10.6 million people had contracted TB by 2022. China has a significant TB burden, with 0.75 million TB cases reported in 2022^3^.

Effective treatment is critical to limit TB spread and lower mortality rates. The coexistence of TB with other chronic diseases has attracted increasing attention in recent years, as it may result in a greater likelihood of mortality and treatment failures^4^. Hyperglycemia is an important risk factor for the development of active TB^5^. Hyperglycemia has been reported to be very frequent in low and middle-income countries where TB is also endemic and prevalent^6–8^. A Peruvian study showed that the prevalence of diabetes mellitus (DM) and prediabetes (PDM) among TB patients was 13.97% and 30.88%, respectively^9^. Another study from South India found that over 54% of patients with TB had DM, and 21% had PDM^10^. A Chinese community survey found that the prevalence of hyperglycemia in patients with TB was 26.5%^11^.

Hyperglycemia weakens the immune system and impairs the body’s ability to effectively respond against *M. tuberculosis*. This weakened immune response may result in delayed clearance of bacteria, prolonged infectivity, and an increased risk of TB relapse^12,13^. Meanwhile, Hyperglycaemic conditions disrupt the delicate balance between pro-inflammatory and anti-inflammatory cytokines, creating an unfavorable immune environment for controlling TB^5,14^. Furthermore, some research indicates that hyperglycemia might have an impact by affecting drug absorption and metabolism of anti-tuberculosis drugs in blood^15^. Therefore, poorly managed blood glucose levels may be associated with an increased risk of TB development^16^. Previous studies have revealed that TB in patients with hyperglycemia may present with different clinical manifestations than in those without hyperglycemia. These distinctions include increased TB symptoms, lung involvement, and bacterial burden^17,18^. However, these results have not been consistently reported. Most previous studies either lacked a follow-up study to determine the causal relationship between hyperglycemia and TB treatment effects or were limited by their small sample size.

The coexistence of TB and hyperglycemia has emerged as a major health concern. The bidirectional relationship between these two diseases poses significant diagnostic, treatment, and disease management-related challenges^19^. Understanding the influence of hyperglycemia on the treatment response of patients with TB is crucial for improving patient care and developing effective management strategies. Our cohort study investigated the prevalence of hyperglycemia in patients with pulmonary TB (PTB) and the effects of hyperglycemia on treatment outcomes of PTB in Shandong Province, China.

## Materials and methods

### Study design and population

We recruited 791 patients who had been diagnosed with active PTB from a city-level TB-specialized hospital in Weifang between 2019 and 2022. The inclusion criteria were as follows: (1) patients who were recently diagnosed with PTB using a combination of sputum smear, computed tomography (CT) scan, and clinical symptoms (*e.g.* cough, sputum production, hemoptysis, fever, chest pain, fatigue, night sweats, and anorexia)^20^; (2) patients aged ≥ 18 years; (3) patients who agreed to sign the informed consent form; and (4) patients who received standard anti-tuberculosis treatment. Exclusion criteria were as follows: (1) patients with hematogenous disseminated PTB, tuberculous pleurisy, and other extrapulmonary TB; (2) patients who were pregnant or lactating; and (3) patients who had other diseases, including cardiovascular diseases, respiratory diseases, cancer, HIV, or neurodegenerative diseases.

### Diagnostic criteria for hyperglycemia

Hyperglycemia screening was based on the WHO criteria for the classification of glucose tolerance based on fasting plasma glucose (FPG). After fasting overnight under the supervision of nurses or family members, venous blood was collected the next morning and FPG levels were measured by glucose oxidase method. Patients with FPG levels ≥ 6.1 mmol/L were diagnosed with hyperglycemia^21^. Fasting blood glucose was measured at least twice in patients with PTB before anti-tuberculosis treatment.

### Procedure

Patient data such as weight and height were measured, and demographic information, including age, sex, education level, area of residence, marital status, smoking, and drinking, was collected using a standard questionnaire upon admission of patients at the hospital. All patients were followed up for 2 months during the intensive anti-tuberculosis therapy phase. All patients received the same standard anti-tuberculosis regimen of daily rifampicin (RFP), isoniazid (INH), pyrazinamide (PZA), and ethambutol (EMB) combination for 2 months^22^. The dosage for INH, RFP, PZA, and EMB were 0.3, 0.45, 1.5, and 0.75 g/d, respectively. In reality, dosages could be adjusted according to the individualization. The signs and symptoms of patients, including cough, sputum production, hemoptysis, chest pain, fever, fatigue, night sweats, appetite loss, and body mass index (BMI) were obtained monthly by questionnaire or physical measurement. The severity of PTB symptoms and signs in patients was assessed using the TB score^23,24^. The presence or absence of each of the first eight signs and symptoms scored one point or no point, respectively. A BMI of less than 16 kg m^−2^ received two points, a BMI of 16–18 kg m^−2^ received one point, and a BMI of more than 18 kg m^−2^ received no point. The range of the TB scores was 0–10. Patients were categorized into two groups based on the TB score: patients with < 4 and patients with ≥ 4 points. Adverse gastrointestinal symptoms, such as nausea, emesis, diarrhea, abdominal distension, and constipation, were recorded monthly using a questionnaire^25^.

Sputum samples were collected and tested for the presence and abundance of acid-fast bacilli (AFB); the presence or absence of AFB was classified as positive or negative, respectively. CT scans of the patients’ chests were collected once before treatment and once at the end of the second month of treatment, both sides of the lung lobes were divided into 5 lung segments, including apical segment or posterior segment, anterior segment of upper lobe, middle segment, dorsal segment of lower lobe, and basal segment. Two radiologists and specialists evaluated the number of lesions (*e.g.* patchy, stripe, nodular, and calcified), cavities, and infiltrations involving the lung segments.

### Statistical analysis

Prevalence rates closely related to the purpose of the study were selected to calculate the sample size. The incidence of pulmonary cavity in patients with hyperglycemic PTB and non-hyperglycemic PTB was 29% and 23%, respectively. Assuming 90% power and accounting for 20% missing data, the minimum sample size required was 666. Statistical analyses were performed using SPSS version 26.0 (IBM Corp, Armonk, NY, USA). All quantitative data that were normally distributed are expressed as mean ± standard deviation (SD). The distributions of categorical variables such as sex, education level, area of residence, marital status, BMI, smoking, and drinking are presented as frequencies and percentages. Intergroup differences were tested using a t-test for numerical data and a Chi-square test or Fisher’s exact t-test for categorical data. A generalized estimating equation was used to analyze the effects of hyperglycemia on the clinical manifestations, lung lesions, gastrointestinal adverse reactions, and sputum smear results at baseline, month one, and month two. Both univariate and multivariate analyses were used, and the multivariate analysis model was adjusted for potential confounding factors, including age, sex, education level, area of residence, marital status, BMI, smoking, and drinking. Adjusted OR and 95% CI were reported to indicate the strength and direction of associations. Statistical significance was set at p < 0.05. significant.

### Ethics approval and consent to participate

This study was approved by the Medical Ethics Committee of the Qingdao Municipal Center for Disease Control and Prevention and followed the Declaration of Helsinki. Informed consent was obtained from each patient, and all data were kept confidential throughout the study.

## Results

**Figure 1:**
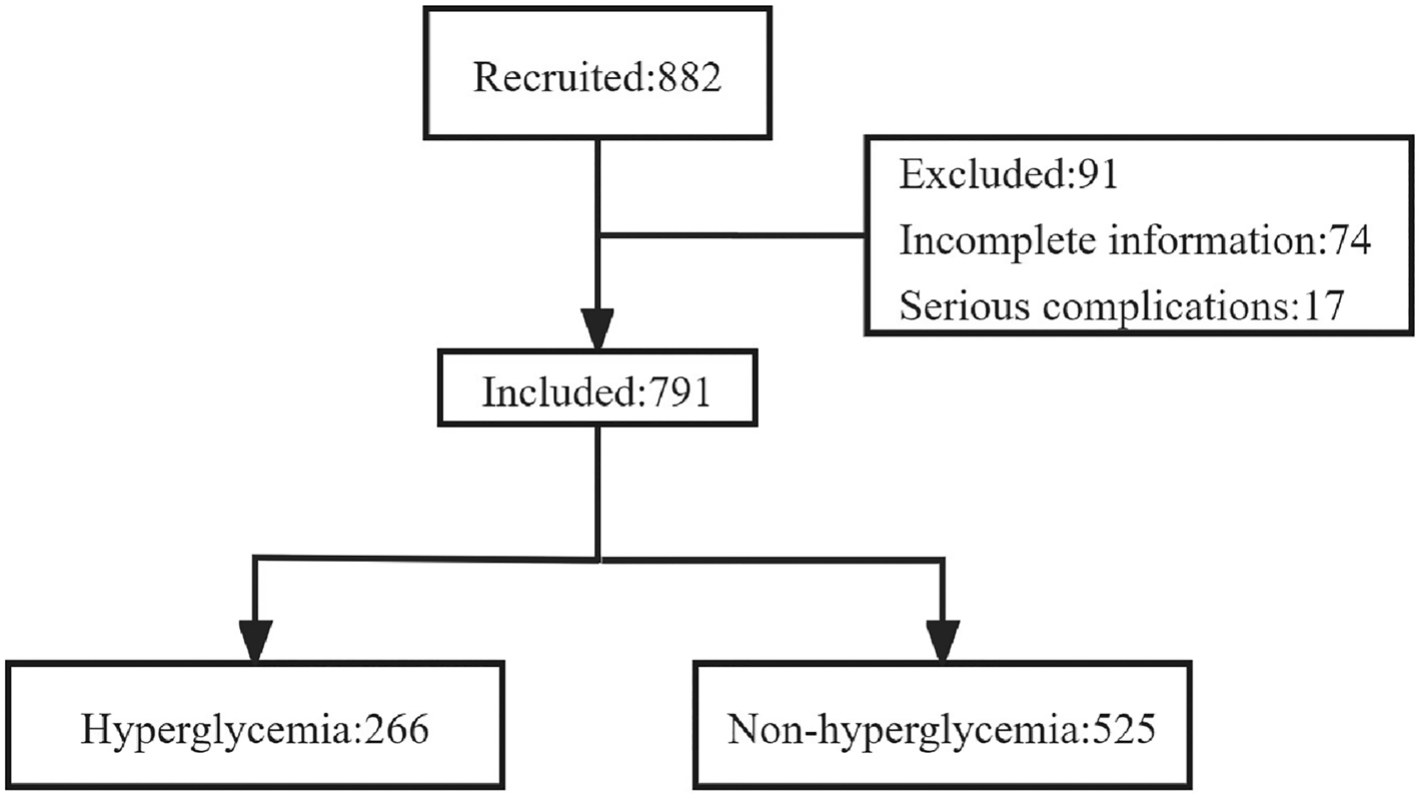
The trial flowchart.

The incidence of symptoms closely related to the purpose of the study was selected to calculate the sample size. The calculation formula of sample size is as follows: A total of 882 patients with PTB were recruited from a city-level TB-specialized hospital in Weifang City, Shandong Province, China (Fig. 1). Among them, 74 did not have complete clinical symptoms or baseline information, and 17 had severe complications. Therefore, 791 patients were included in this study. There were 266 patients (33.6%) who exhibited hyperglycemia. Smoking and drinking habits were similar between the hyperglycemic and non-hyperglycemic groups. However, patients with hyperglycemia were predominantly male, older, married, rural residents, and had lower educational levels. Furthermore, patients with hyperglycemia had a higher BMI than those without hyperglycemia (Table 1).

**Table 1:** Baseline characteristics for the included pulmonary TB patients.

Characteristics	Total (N = 791)	Non-hyperglycemia (N = 525)	Hyperglycemia (N = 266)	P-value
Mean (± SD) Age	42.44 ± 17.63	37.51 ± 16.51	52.17 ± 15.64	< 0.001
Sex				< 0.001
Women	254 (32.1)	194 (37.0)	60 (22.6)	
Men	537 (67.9)	331 (63.0)	206 (77.4)	
Residence				0.002
Urban	265 (33.5)	195 (37.1)	70 (26.3)	
Rural	526 (66.5)	330 (62.9)	196 (73.7)	
Education				< 0.001
Illiteracy	31 (3.9)	11 (2.1)	20 (7.5)	
Primary and junior high school	344 (43.5)	196 (37.3)	148 (55.6)	
Senior and technical secondary school	309 (39.1)	225 (42.9)	84 (31.6)	
Diploma or higher	107 (13.5)	93 (17.7)	14 (5.3)	
Marital status				< 0.001
Live alone	256 (32.4)	218 (41.5)	38 (14.3)	
Married	535 (67.6)	307 (58.5)	228 (85.7)	
BMI (kg/m^2^)				0.001
< 18.5	193 (24.4)	141 (26.9)	52 (19.5)	
18.5–23.9	474 (59.9)	318 (60.6)	156 (58.6)	
≥ 24.0	124 (15.7)	66 (12.6)	58 (21.8)	
Smoking				0.804
Yes	53 (6.7)	36 (6.9)	17 (6.4)	
No	738 (93.3)	489 (93.1)	249 (93.6)	
Drinking				0.933
Yes	35 (4.4)	23 (4.4)	12 (4.5)	
No	756 (95.6)	502 (95.6)	254 (95.5)	

Unless indicated otherwise, data are presented as n (%).

Before treatment, patients with TB and hyperglycemia showed a significantly higher incidence of a TB score > 4, cough, fatigue, and night sweats compared to patients with TB but without hyperglycemia (Table 2). Patients with TB and hyperglycemia also experienced significantly rates of cough, sputum production, and night sweats after two months of treatment (Table 2). After adjusting for multiple confounding factors (Table 3), generalized estimating equation analysis revealed that the risk of elevated TB scores was 1.569 times higher in patients with hyperglycemia than in those without (95% CI 1.040–2.369). The risk of major symptoms such as cough and night sweats were 1.332 times (95% CI 1.050–1.690) and 1.694 times (95% CI 1.228–2.335) higher, respectively, in patients with TB with hyperglycemia compared to those without hyperglycemia (Table 3).

**Table 2:** Comparison of TB score and incidence of clinical symptoms between two groups of patients.

Variables	Baseline		*P*	2-month		*P*
	Non-hyperglycemia (N = 525)	Hyperglycemia (N = 266)		Non-hyperglycemia (N = 509)	Hyperglycemia (N = 242)	
TB score ≥ 4	65 (12.4)	54 (20.3)	0.004	11 (3.1)	3 (1.7)	0.410
Cough	331 (63.0)	190 (71.4)	0.019	127 (25.0)	88 (36.4)	0.001
Sputum production	245 (46.7)	138 (51.9)	0.166	76 (14.9)	56 (23.1)	0.006
Hemoptysis	46 (8.8)	33 (12.4)	0.106	6 (1.2)	5 (2.1)	0.345
Fever	123 (23.4)	63 (23.7)	0.936	8 (1.6)	6 (2.5)	0.397
Chest pain	80 (15.2)	37 (13.9)	0.619	40 (7.9)	23 (9.5)	0.462
Fatigue	114 (21.7)	85 (32.0)	0.002	123 (24.2)	63 (25.9)	0.611
Night sweats	79 (15.0)	56 (21.1)	0.034	39 (7.7)	32 (13.2)	0.015
Loss of appetite	98 (18.7)	65 (24.4)	0.058	57 (11.1)	28 (11.3)	0.934

**Table 3:** Multivariable analysis of the effect of Hyperglycemia on TB score and clinical symptoms during anti-tuberculosis treatment in patients.

Dependent variables	Crude model^†^		Model 1^‡^	
	OR (95%CI)	*p*^§^	OR (95%CI)	*p*^§^
TB score ≥ 4	1.558 (1.094,2.220)	0.014	1.569 (1.040,2.369)	0.032
Cough	1.537 (1.241,1.903)	< 0.001	1.332 (1.050,1.690)	0.018
Sputum production	1.430 (1.144,1.786)	0.002	1.170 (0.916,1.496)	0.208
Hemoptysis	1.681 (1.087.2.599)	0.020	1.276 (0.784,2.077)	0.327
Fever	1.155 (0.844,1.579)	0.368	1.279 (0.917,1.786)	0.148
Chest pain	0.981 (0.699,1.378)	0.913	1.333 (0.918,1.937)	0.131
Fatigue	1.317 (1.038,1.672)	0.023	1.214 (0.937,1.573)	0.142
Night sweats	1.617 (1.214,2.153)	0.001	1.694 (1.228,2.335)	0.001
Loss of appetite	1.105 (0.829,1.473)	0.496	0.884 (0.637,1.227)	0.462

*OR* odds ratio, *CI* confidence interval

^†^Crude model unadjusted

^‡^Model 1 adjusted for age, sex, education level, area of residence, marital status, BMI, smoking, and drinking

^§^Generalized estimation equation

The number of lung segments involved in cavitation was considerably higher in patients with TB and hyperglycemia than in patients without hyperglycemia before and after two months of treatment (Table 4). However, the generalized estimating equation results showed that hyperglycemia did not significantly affect pulmonary lesions in patients with TB (Table 5).

**Table4:** Comparison of pulmonary lesions between two groups of patients.

Variables	Baseline			2-month		
	Non-hyperglycemia (N = 406)	Hyperglycemia (N = 217)	*P*	Non-hyperglycemia (N = 168)	Hyperglycemia (N = 79)	*P*
Number of lung segments involved in the lesion			0.074			1.000
0	5 (1.2)	2 (0.9)		1 (0.6)	0 (0.0)	
1 ~ 3	285 (70.2)	134 (61.8)		115 (68.5)	54 (68.4)	
4 ~ 10	116 (28.6)	81 (37.3)		52 (31.0)	25 (31.6)	
Number of lung segments involved in cavitation			0.046			0.011
0	257 (63.3)	116 (53.5)		115 (68.5)	39 (49.4)	
1 ~ 3	141 (34.7)	97 (44.7)		50 (29.8)	38 (48.1)	
4 ~ 10	8 (2.0)	4 (1.8)		3 (1.8)	2 (2.5)	
Number of lung segments involved in infiltration			0.407			0.776
0	255 (62.8)	129 (59.4)		131 (78.0)	59 (74.7)	
1 ~ 3	114 (28.1)	61 (28.1)		26 (15.5)	15 (19.0)	
4 ~ 10	37 (9.1)	27 (12.4)		11 (6.5)	5 (6.3)

**Table 5:** Multivariable analysis of the effect of hyperglycemia on the degree of pulmonary lesions during anti-tuberculosis treatment in patients with TB.

Dependent variables	Crude model^†^		Model 1^‡^	
	OR (95%CI)	*p*^§^	OR (95%CI)	*p*^§^
Number of lung segments involved in the lesion	1.410 (1.010,1.970)	0.044	0.996 (0.674,1.471)	0.982
Number of lung segments involved in cavitation	1.018 (0.339,3.053)	0.975	1.021 (0.325,3.202)	0.972
Number of lung segments involved in infiltration	1.448 (0.873,2.402)	0.152	1.239 (0.687,2.234)	0.476

*OR* odds ratio, *CI* confidence interval

^†^Crude model unadjusted

^‡^Model 1 adjusted for age, sex, education level, area of residence, marital status, BMI, smoking, and drinking.

^§^Generalized estimation equation

The sputum results for mycobacterial analysis during intensive anti-TB treatment in both groups of TB patients are shown in Table 6. The rate of positive sputum tests before treatment, and at the end of the second month of treatment was substantially higher in patients with TB with hyperglycemia than in those without hyperglycemia (Table 6). Furthermore, the generalized estimating equation analysis showed that patients with hyperglycemia had a 1.941-fold greater chance of positive sputum test positive occurrence (95% CI 1.382–2.727) than patients without hyperglycemia (Table 7).

**Table 6:** Comparison of sputum mycobacterial results between the two groups of patients.

Variables	Baseline			2-month		
	Non-hyperglycemia (n = 370)	Hyperglycemia (n = 192)	*P*	Non-hyperglycemia (n = 353)	Hyperglycemia (n = 170)	*P*
Sputum Smear			0.001			< 0.001
Positive	133(35.9)	97(50.5)		31(8.8)	47(27.6)	
Negative	237(64.1)	95(49.5)		322(91.2)	123(72.4)

**Table 7:** Multivariable analysis of the effect of hyperglycemia on sputum mycobacterial results during anti-tuberculosis treatment in patients with TB.

Dependent variables	Crude model^†^		Model 1^‡^	
	OR (95%CI)	*p*^§^	OR (95%CI)	*p*^§^
Sputum Smear	2.411 (1.787,3.253)	< 0.001	1.941 (1.382,2.727)	< 0.001

*OR* odds ratio, *CI* confidence interval.

^†^Crude model unadjusted.

^‡^Model 1 adjusted for age, sex, education level, area of residence, marital status, BMI, smoking, and drinking.

^§^Generalized estimation equation.

The incidence of adverse gastrointestinal reactions during intensive anti-TB treatment in both groups of patients with TB is shown in Table 8. The findings revealed that patients with TB with hyperglycemia had a higher incidence of abdominal distention before treatment, and a significantly higher incidence of vomiting, diarrhea, and constipation at the end of the second month of treatment compared to patients without hyperglycemia (Table 8). A similar analysis explored the association between hyperglycemia and adverse gastrointestinal reactions. We found that hyperglycemia was associated with a higher risk of vomiting, abdominal distension, and constipation during treatment than in patients without hyperglycemia. The odds ratio (OR) of vomiting was 2.738 (95% CI 1.041–7.198), the OR of abdominal distension was 2.230 (95% CI 1.193–4.171), and the OR of constipation was 2.372 (95% CI 1.442–3.902) (Table 9).

**Table 8:** Comparison of the incidence of gastrointestinal adverse reactions in the two groups.

Variables	Baseline		*P*	2-month		*P*
	Non-hyperglycemia (n = 525)	Hyperglycemia (n = 266)		Non-hyperglycemia (n = 509)	Hyperglycemia (n = 242)	
Nausea	32 (6.1)	20 (7.5)	0.445	36 (7.0)	21 (8.8)	0.398
Vomiting	11 (2.1)	7 (2.6)	0.633	3 (0.6)	5 (2.2)	0.046
Diarrhea	14 (2.7)	6 (2.3)	0.728	7 (1.3)	9 (4.0)	0.021
Abdominal Distension	7 (1.3)	13 (4.9)	0.003	19 (3.6)	10 (4.4)	0.634
Constipation	9 (1.7)	10 (3.8)	0.076	16 (3.0)	29 (12.8)	< 0.001

**Table 9:** Multivariable analysis of the effect of hyperglycemia on gastrointestinal adverse reactions during anti-tuberculosis treatment in patients with TB.

Dependent variables	Crude model^†^		Model 1^‡^	
	OR (95%CI)	*p*^§^	OR (95%CI)	*p*^§^
Nausea	1.199 (0.810,1.774)	0.365	1.446 (0.927,2.256)	0.104
Vomiting	1.942 (0.943,3.998)	0.072	2.738 (1.041,7.198)	0.041
Diarrhea	1.468 (0.791,2.723)	0.223	1.493 (0.733,3.038)	0.269
Abdominal distension	1.683 (0.972,2.914)	0.063	2.230 (1.193,4.171)	0.012
Constipation	2.824 (1.790,4.455)	< 0.001	2.372 (1.442,3.902)	0.001

*OR* odds ratio, *CI* confidence interval.

^†^Crude model unadjusted.

^‡^Model 1 adjusted for age, sex, education level, area of residence, marital status, BMI, smoking, and drinking.

^§^Generalized estimation equation.

## Discussion

Our study showed that hyperglycemia was prevalent among patients with TB. In patients with TB, hyperglycemia was associated with an increased risk of severe clinical symptoms (as shown by higher TB scores), positive sputum smears, and adverse gastrointestinal effects. Thus, our findings may imply that hyperglycemia may significantly affect the effectiveness of anti-tuberculosis treatment.

After 2 months of TB treatment, hyperglycemia was positively correlated with positive sputum smear results, even after controlling for possible confounding factors. In sputum smear tests, hyperglycemia is an independent risk factor associated with increased AFB. Immunological suppression caused by hyperglycemia may be attributed to an increased smear-positivity rate. Hyperglycemia impairs the immunological response and causes the malfunction of immune cells, such as macrophages, monocytes, and lymphocytes, which are necessary for controlling TB infection^26–28^. Hyperglycemic conditions can inhibit the phagocytic activity of macrophages, thereby limiting their ability to engulf and eliminate AFB. Interferon-γ enhances nitric oxide-dependent intracellular killing capacity in macrophages. Hyperglycemia may impair T-cell interferon production and T-cell development, function, and proliferation, leading to a diminished immune response and allowing AFB to replicate and persist, resulting in a higher AFB smear positivity^29^. In addition, hyperglycemia causes dysfunction of polymorphonuclear leukocytes, thereby reducing their bactericidal activity^30^. However, there is a complex relationship between hyperglycemia and AFB smear positivity in patients with TB, and further research is needed to understand the underlying mechanisms.

In addition to immune function suppression, the increased severity of TB may also result from the increased severity of the sustained inflammatory response that occurs in the body in the hyperglycemic state, including the elevation of systemic inflammatory markers and the activation of inflammatory cells^14,31^. We found that, after adjusting for possible confounders, patients with hyperglycemia had a higher risk of elevated TB scores than those without hyperglycemia, which is consistent with the findings of a study from Indonesia^32^. Coughing and night sweats are major symptoms that occur more frequently in individuals with hyperglycemia than in those without hyperglycemia. Cough is one of the most common symptoms of TB, and patients are often more prone to cough symptoms owing to compromised immune function^33^. Night sweats may be caused by an increase in the release of pro-inflammatory factors, such as tumor necrosis factor and interleukin-6, which influence the thermoregulatory centers of the brain and cause excessive sweating, particularly at night^34–36^.

Previous studies have shown that patients with TB with hyperglycemia presented with cavities, alveolar infiltrates, and fibrous tracts more frequently than those with normoglycemia^6,37^. However, the results of this study are contrary, and no significant effect of hyperglycemia on pulmonary lesions in patients with TB was found after controlling for confounding factors. Although, another review investigated the effect of hyperglycemia on the radiological manifestations of TB and found conflicting results^38^.

Our study revealed that patients with TB and hyperglycemia were more likely to experience gastrointestinal side effects, with similar results found in another projected follow-up in Hong Kong^39^. However, in another study from Thailand, hyperglycemia was associated with 2.5- and 3.9-fold more adverse drug reactions during the intensive and continuous treatment phases, respectively, in a crude univariate analysis, but the difference was not statistically significant^40^. The reason why patients with TB patients with hyperglycemia could be prone to adverse gastrointestinal reactions may be due to dysbiosis of the intestinal flora caused by the hyperglycemic state, which reduces the number of beneficial bacteria, such as Lactobacillus and Bifidobacterium, and increases the number of pathogenic bacteria. Dysbiosis can lead to adverse intestinal reactions^41^.

Our study has several strengths. Most previous investigations on the impact of hyperglycemia on TB were constrained by small sample sizes. However, our cohort study included 266 sputum smear-positive TB patients with hyperglycemia and 525 non-hyperglycemic controls, allowing us to draw several significant conclusions. Notable, our statistical approach used a GEE analysis, which is an effective statistical method for analyzing longitudinal data. Issues related to repetitive data measures and missing data were overcome, and the validity of the study results was enhanced. Furthermore, a composite index TB score was utilized to identify the severity of TB symptoms in each participant, and hematological and biochemical measures were low-cost, easy-to-perform and gather methods.

Notably, Kumar's study demonstrated a negative correlation between blood glucose and antituberculosis drug plasma concentrations, suggesting delayed absorption or faster elimination of INH and PZA in the presence of elevated glucose^15^. The blood concentration of anti-tuberculosis drugs is directly related to the efficacy^42,43^, so the insufficient blood concentration may be one of the reasons for the poor effect of anti-tuberculosis treatment caused by hyperglycemia.

However, this study had some limitations. Firstly, in our study, we did not perform 2-h oral glucose tolerance tests (OGTTs). Most participants lived in rural areas and often had a long travel distance to the hospital, making it impractical to perform OGTTs. This may have led to an underestimation of the prevalence of hyperglycemia. Secondly, the study sample was drawn from only one hospital in Weifang, Shandong Province, China, posing geographical limitations. Therefore, our results may not be applicable to other regions or populations. Third, the sample selection for this study was conducted within the hospital; therefore, there may have been a patient selection bias. This may have resulted in an under-representative sample, which may have affected the reliability and generalizability of the results.

## Conclusion

The present study showed a significant association between hyperglycemia and TB. Patients with TB and hyperglycemia are at higher risk of severe clinical manifestations, positive sputum test results, and adverse gastrointestinal effects. These results suggest that the special situation of hyperglycemic patients should be considered in the prevention and treatment of TB for better management and control of the progression of TB.

## Data Availability

The datasets generated and/or analysed during the current study are not publicly available due the database is writing the article but are available from the corresponding author on reasonable request.

## Abbreviations

PTB: Pulmonary tuberculosis
CT: Computed tomography
GEE: Generalized estimating equation
TB: Tuberculosis
OR: Odds ratio
WHO: World Health Organization
DM: Diabetes mellitus
PDM: Prediabetes
FPG: Fasting plasma glucose
BMI: Body mass index
AFB: Acid-fast bacilli
SD: Standard deviation
OGTTs: Oral glucose tolerance tests
TDM: Therapeutic drug monitoring
RFP: Rifampicin
INH: Isoniazid
PZA: Pyrazinamide
EMB: Ethambutol
